# Improving Mental Health Care in Developing Countries Through Digital Technologies: A Mini Narrative Review of the Chilean Case

**DOI:** 10.3389/fpubh.2019.00391

**Published:** 2019-12-20

**Authors:** Graciela Rojas, Vania Martínez, Pablo Martínez, Pamela Franco, Álvaro Jiménez-Molina

**Affiliations:** ^1^Hospital Clínico, Universidad de Chile, Santiago, Chile; ^2^Millennium Nucleus to Improve the Mental Health of Adolescents and Youths (Imhay), Santiago, Chile; ^3^Millennium Institute for Research in Depression and Personality (MIDAP), Santiago, Chile; ^4^Millennium Nucleus in Social Development (DESOC), Santiago, Chile; ^5^Centro de Medicina Reproductiva y Desarrollo Integral del Adolescente (CEMERA), Facultad de Medicina, Universidad de Chile, Santiago, Chile; ^6^Doctoral Program in Psychotherapy, Pontificia Universidad Católica de Chile, Santiago, Chile

**Keywords:** telemedicine, e-mental health, Internet, digital technologies, primary health care, developing countries

## Abstract

The uneven distribution of mental health resources contributes to the burden of mental disorders in vulnerable groups, especially in developing countries. Internet-based interventions and digital technologies can contribute to reducing the gap between high prevalence of mental disorders, demand for treatment, and access to mental health care, thereby reducing inequities in mental health. This mini review summarizes the current state of the field of e-mental health research in Chile, showing its progress, limitations, and challenges. Internet-based interventions are at an early stage of development in Chile. The interventions included are heterogeneous in terms of participants (e.g., secondary students, patients, healthcare professionals) and contexts (e.g., rural, urban, schools, primary health care), aims, and modalities (e.g., website, online games). While these studies confirmed the feasibility of Internet-based interventions, the shortage of studies on effectiveness and cost-effectiveness makes it difficult to disseminate and scale up these Internet-based programs. However, the growing amount of knowledge accumulated in the Chilean context could guide practices in other developing countries for supporting the mental health of underserved populations.

## Introduction

Developing countries are facing the impact of mental health problems while confronted with limited resources and inequities in access to mental health care (1–5). The use of Internet and digital technologies has the potential to address these gaps, facilitating the development of more equitable models of care in a variety of contexts (6–8).

Internet and digital technologies could be a powerful strategy for the delivery of mental health services in low-resource settings. Although Internet-based and digital interventions have shown their potential benefits in developed countries (8–10), there is a lack of studies in developing countries. In particular, randomized controlled trials (RCTs) investigating the effectiveness of Internet-based and digital interventions in developing countries is lacking (11–13). It is, therefore, crucial to closely study e-mental health experiences in developing countries, in order to learn from their successes and limitations.

Chile is a developing Latin American country with a high prevalence of mental disorders and marked socio-economic and geographical inequities in access to health care (14–17). Since the 1990s, Chile has developed community-based mental health services throughout the country to reduce the gap between mental health needs and access to treatment (18). The public health system, which serves approximately 70 per cent of the population including the most at-risk groups in Chile, between the years 2000 and 2010 increased the amount of human resources for specialized mental health care and the number of mental health centers, especially in primary health care (18, 19). In addition, in 2006 Chile introduced a policy that guarantees access to and quality of care, as well as financial protection for prioritized diseases (20), including four mental pathologies (depression in people over the age of 15, bipolar disorder, addictions in people under 20 years of age, and first episode of schizophrenia). In this way, Chile provides one of the first examples of an evidence-based depression intervention being scaled up in resource-constrained settings (21, 22).

However, after 25 years of relatively successful mental health policies, mental health in Chile still presents serious deficiencies and continues to be considered a great challenge for public health (23). There is still a striking imbalance between government spending on mental health and the related disease burden in Chile (4). Thus, the treatment gap remains high: only 38.5% of those with a diagnosis receive any type of mental health service (17), and there are long waiting lists for psychological treatments in primary health care settings (23, 24). This gap is unequally distributed among social strata. Multiple socioeconomic and territorial inequalities persist, with a high concentration of specialized mental health services in the capital city (18).

Digital technologies are highly utilized across all social strata in Chile (25). This high level of digital connectivity (26) has recently facilitated the development of e-mental health interventions in an effort to improve access to treatment for different populations across the country and to utilize preventative methods.

The aim of this article is to provide a description of the use of Internet and digital technology in mental health through a narrative mini-review of studies conducted in Chile. The lessons learned in the Chilean context can inform the delivery of mental health services in low-resource settings with access to Internet and digital technologies.

## Methods

We conducted a literature search of available source describing the use of Internet and digital technologies in mental health in Chile. Anticipating a small number of randomized clinical trials, we decided to do a narrative mini-review of the literature. A systematic or scoping review method was not used because it would have required greater consideration of intervention effectiveness. A mini-review allowed for greater discussion of important areas in which we believe that Internet and digital technologies could yield considerable gains toward addressing mental disorders in developing countries.

### Procedure and Literature Search Strategy

We searched for published papers, written in English or Spanish, indexed from inception to November 2018 in PubMed, Embase, and SciELO databases. Given the shortage of studies, we searched for study protocols and unpublished data through contact with national experts in the field. Search strategies are detailed in the Supplementary Material.

The following study eligibility criteria were applied: (1) articles that relate the use of mental health interventions based on or supported by Internet and digital technologies; (2) feasibility and acceptability studies, pre-post evaluation (with no control group) and RCTs; (3) studies conducted in Chile.

### Study Selection and Data Collection Process

**Figure 1:**
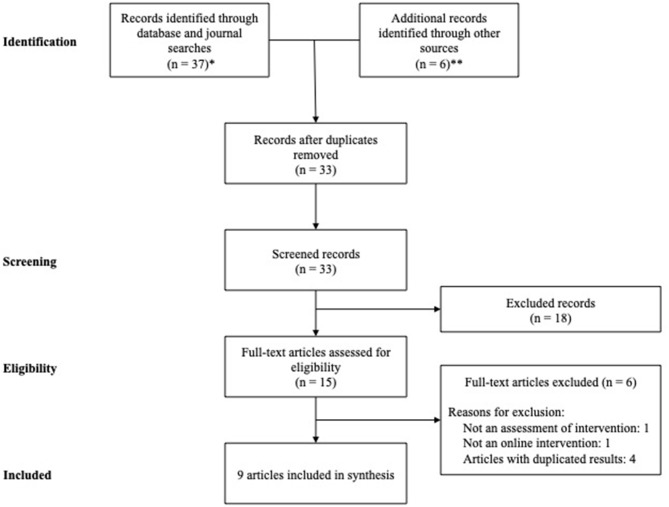
PRISMA Flow diagram. *PubMed: 20 records; Embase: 12 records; Scielo: 5 records. **Presentations in scientific congresses: 2 presentations; Personal contact: 3 drafts of preliminary results; Reported in other review: 1.

Study selection and data collection were carried out by teams of two reviewers in an independent manner (GR and VM, AJ-M and PF). Study records were compiled and duplicates were removed. The study selection process was documented using the PRISMA Extension for Scoping Reviews (PRISMA-ScR) flow diagram (Figure 1) in order to achieve a correct organization of the procedure (27). If the information was insufficiently detailed in the original article, we contacted the corresponding author asking for the preliminary study results. Narrative techniques were the selected approach for data analysis and synthesis, with due emphasis on study characteristics.

## Results

After the full-text eligibility assessment, we found three RCTs (28–30), three acceptability and/or feasibility studies [(31, 32); Martínez et al. under review], and three on-going studies without published results [(33, 34); Irarrázaval et al. under review]. The responsible researcher from three studies (Gaete et al., under review; Martínez et al., under review; Irarrázaval et al., under review) provided details of it and its preliminary results. The main characteristics of each study are presented in Table 1.

**Table 1:** Description of studies.

**Study**	**Population setting/N**	**Condition of interest/age group**	**Outcome measures**	**Intervention/time**	**Supervision/** **Contact method**	**Design/Control group (CG)**	**Measurement time**	**Follow-up**
Rojas et al. (33) [protocol]	Public primary care centers/ *N =* 434	Depressive symptomatology/18-65 years old	Primary outcome: depressive symptoms (PHQ-9) Secondary outcomes: health-related quality of life; service use; patient satisfaction; psychotherapy outcomes (OQ 45.2)	16-h training program for primary care teams (detection, diagnosis, and treatment of depression)	Research team/web-based platform and call center	Two-arm, single-blind CG: usual care (*n =* 217)	Baseline and follow-up	3, 6, and 12 months after baseline assessment
Carrasco (31)	Private and public outpatient health centers patients and therapists/ Patients *N =* 15; Therapists *N =* 5	Mild or moderate depression/female adolescents (12–18 years old)	Acceptability scale Interviews with therapists	Online video game	Research team/web-based platform (patients); in person (therapists)	Acceptability study CG: No control group	Post-intervention	No
Espinosa et al. (32)	Private outpatient clinic/ *N =* 35	Major depression (discharged patients)/ 18-65 years old	Acceptability and satisfaction questionnaire Semi-structured interviews	Web-based program for supporting and monitoring of depressive patients after treatment; 9 months	Research team/web-based platform and e-mail	Feasibility and acceptability study CG: No control group	Post-intervention	No
Gaete et al. (30) [protocol]	Public primary schools/ *N =* 4485	Bullying victims and perpetrators/5th and 6th grades (10-12 years old)	Primary outcomes: bullying and victimization (Revised Olweus Bully/Victim Questionnaire, OBVQ) Secondary outcomes: psychosocial adjustment, psychological sense of school membership, academic performance	Ten 2-h lessons delivered by trained teachers, posters encouraging to support victims, discussion groups, online game; 1 year Partial KiVa group: without online game (*n =* 1495)	Research team/in person	Three-arm, single-blind (blinded only to the outcome evaluator), cluster RCT CG: usual management for bullying (*n =* 1495)	Baseline and post-intervention	No
Rojas et al. (28)	Community hospitals located in rural areas/ *N =* 250	Major depressive disorder/18-70 years old	Primary outcome: depressive symptoms (Beck Depression Inventory, BDI-I) Secondary outcomes: health-related quality of life; treatment adherence to antidepressants; service use; patient satisfaction	Remote supervision by a psychiatrist through an electronic platform and/or telephone; 6 months	Research team/online and phone call	Nonrandomized, open-label (blinded outcome assessor) trial, two-arm CG: usual care (*n =* 139)	Baseline and follow-up	3 and 6 months after assignment
Martínez et al. (29)	Public primary care centers/ *N =* 143	Major depressive disorder/13-19 years old	Primary outcome: depressive symptoms (Beck Depression Inventory, BDI) Secondary outcomes: health-related quality of life; patient adherence and satisfaction; clinician satisfaction	Remote collaborative depression care (primary health care teams received remote supervision by a psychiatrist through a shared electronic health record and phone patient monitoring); 3 months	Research team/online and phone call	Cluster randomized, assessor-blind trial, two-arm CG: enhanced usual care (*n =* 78)	Baseline and follow-up	12 weeks
Mascayano et al. (34) [protocol]	Public high-schools/ *N =* 428	Suicidal ideation/14-18 years old	Primary outcome: Suicidality (Okasha Questionnaire) Secondary outcomes: impulsivity; self-esteem; stigma-discrimination; depressive symptoms; anxiety; utility and functionality	Project Clan (web-based platform and mobile app to cultivate a community to promote protective psychological and social factors); 3 months	Psychologist as online counselor/web platform	Two-arm, cluster RCT; participative approach (peer-adolescent) CG: adolescents without intervention (*n =* 214)	Baseline, post-intervention, and follow-up	2 month
Martínez et al. (under review)	Public and semi-private high-schools from Chile and Colombia/ *N =* 199	Moderate depressive symptomatology/14-17 years old	Primary outcome: depressive symptoms (Patient Health Questioner, PHQ-9) Secondary outcomes: automatic thoughts, social problem-solving, health-related quality of life	Stepped-care program according to PHQ-9 score: psychoeducational information, symptom monitoring with personalized automatic feedback, group forum and chat, reference to face-to-face attention if required; 12 weeks, with subsequent bi-monthly reinforcement sessions	Research team/email and chat	Feasibility and acceptability study CG: No control group	Baseline, post-intervention and follow-up	6 months after intervention
Irarrázaval et al. (under review)	Public primary health care center	Children and adolescents living in substitute care facilities	Primary outcome: case resolutions (e.g. number of cases with positive resolution) Secondary outcomes: usefulness and acceptability	90-minute online mental health supervision (diagnostic assistance, management guidance, assessment in referral to specialized services); twice a month for 6 months	Psychiatrist/ videoconference and online shared clinical record system	Quasi-experimental design and acceptability study CG: No control group	Pre-post test	No

### Remote Collaborative Depression Care Programs

Rojas et al. (28) tested the feasibility, acceptability, and effectiveness of a remote collaborative depression care (RCDC) intervention for patients living in rural areas. The RCDC methods used Web-based shared electronic health records (SEHR) between primary care teams and a specialized mental health team, remote supervision by psychiatrists through the SEHR and/or telephone, and telephone monitoring of patients. Once a week, the specialized mental health team reviewed data entered into the SEHR by the primary care team, and provided remote assistance by entering suggestions into the platform, and in special cases by giving indications over the telephone. Their results showed that the RCDC program achieved higher user satisfaction (odds ratio [OR] 1.94, 95% CI 1.25–3.00) and better treatment adherence rates (OR 1.81, 95% CI 1.02–3.19) at 6 months compared to usual care. There were no statically significant differences in depressive symptoms between the RCDC program and usual care, but a trend was observed in favor of the intervention group. Significant differences between groups in favor of the RCDC program were observed at 3 months for mental health-related quality of life (beta 3.11, 95% CI 0.19–6.02).

Martínez et al. (29) tested the feasibility, acceptability, and effectiveness of a RCDC intervention for adolescents with depression. The intervention group received periodic remote supervision by psychiatrists located in Santiago, through SEHR and phone patient monitoring. The SEHR functioned as a discussion forum allowing for specialists to assist primary care teams during diagnostic processes and treatment during the acute phase of disorders through personalized, confidential, and real-time interaction. Phone calls to adolescents and his/her primary caregiver included monitoring of different aspects (e.g., adherence to pharmacological treatment). Primary care clinicians were satisfied with the RCDC intervention, valuing the usefulness of receiving timely specialized support. However, there were no significant differences in depressive symptoms or health-related quality of life between groups. The adolescents in the RCDC intervention group were more satisfied with psychological assistance than those in the enhanced usual care group. Satisfaction with psychological care, in both groups, was related to a significant change in depressive symptomatology at 12-weeks follow-up (beta = −4.3, 95% CI −7.2 to −1.3).

### Prevention Programs for Children and Adolescents

In the study of Gaete et al. (30), researchers from Chile and Finland collaborated on adapting Finland's KiVa anti-bullying program (35) for 4th and 5th grade students in Chile and on evaluating its effectiveness in this new context. KiVa is an evidence-based program to prevent bullying and tackle bullying cases. Students in the full and partial KiVa groups received universal actions and indicated actions. Universal actions focused mainly on preventing bullying and were delivered to the students in lessons lead by trained teachers. The indicated actions were intended to be used when a bullying case emerged and to be managed by a designated KiVa team in the school. Half of the schools in the KiVa condition also received an online game, which had the intention to raise awareness of the role of the group in bullying, increase empathy and promote strategies to support victimized peers. The study results showed that KiVa program (with or without the online game) was an effective intervention among 5th graders (initially 4th graders when they enrolled in the study) to reduce self-reported victimization (with game: beta = 0.19 95% CI 0.05 −0.33; without game: beta = −0.28 95% CI −0.43 −0.12) and peer-reported bullying actions (with game: beta = 0.33 95% CI 0.11 −0.55; without game: beta = −0.44 95% CI −0.68 −0.19), but no significant difference was found between the two interventions and the control regarding students reporting being a perpetrator (Gaete et al., under review). The online game did not increase the effectiveness of the intervention. Among 6th graders (initially 5th graders when they enrolled in the study), no significant differences were found between the intervention groups and the control, in all the assessments.

Martínez et al. (under review) conducted a study to evaluate the feasibility and acceptability of a stepped-care Internet-based program for the prevention and early intervention of adolescent depression, called Cuida tu ánimo [Take Care of Your Mood] (CTA). One hundred and ninety-five adolescents from two Chilean schools (*n* = 146) and two Colombian schools (*n* = 53) interacted with the program through monitoring and feedback messages, which were delivered every 2 weeks, and a website that allowed them to access psychoeducational content. In addition, the website provided emergency information and allowed users to contact a specialist via online chat appointments. Adolescents with severe depressive symptoms or suicidal risk were invited to participate in an online chat appointment, a phone session, or a face-to-face assessment with a mental health professional. Seventy percent answered at least three monitoring e-mails while participating in the program. The number of responses decreased after the 4th monitoring e-mail. During the 3-month program, three participants accessed an online counseling appointment, one participated in a phone call session, and five had a face-to-face assessment. The results showed that implementation of the CTA program pilot were feasible. However, the authors point out that it is necessary to involve adolescents in the design of the intervention in order to encourage and maintain participation.

### Feasibility and Acceptability Studies for Depression Management

Carrasco (31) studied the acceptability of an online adventure video game as a psychotherapeutic tool for female adolescents in psychotherapy for mild or moderate depression. As part of the game design process, the author conducted focus groups with adolescent females. In the game, players follow the story of a female adolescent, who gets involved in interpersonal situations that require psycho-social reasoning. The scoring system provides cues about positive game behavior in the areas of: recognition and modification of negative cognitive bias; interpersonal skills and interpersonal problem solving; and behavioral activation and a healthy lifestyle. Fifteen adolescent females played the game as suggested by their therapists. Patients could play the game as many times as they wanted. The average playtime was 11:57 min (*SD* = 03:42). Most patients played once, four patients played twice. The majority of patients (*n* = 11) reported positive acceptability rates. This indicates that most of them valued the game and that they thought that they could obtain mental health-related benefits from playing it. The patient's therapists were also interviewed, and all gave positive feedback on the game and felt the game was useful for various reasons (e.g., that it was possible in post-game sessions to relate elements of it with aspects of the patients' lives).

Espinosa et al. (32) studied the feasibility and acceptability of the Chilean version of the Supportive Monitoring and Disease Management over the Internet program [SUMMIT (36)], which was called ASCENSO in Spanish. This program aims to monitor and support patients after being discharged from depression treatment. The monitoring consisted of an online assessment of symptoms every 2 weeks and automated feedback. In cases where a patient reported severe impairment, the ASCENSO team contacted the patient to explore the need for further professional support. In cases where a patient showed moderate impairment, the patient received a recommendation to access a personalized self-care plan on the website. Patients were able to book an online chat counseling appointment with a psychologist at any time. When the program monitoring displayed severe symptomatology impartment, the patient feedback reminded patients of the online chat counseling service. Although only half of the participants actively used the program, most of them displayed a good level of acceptance. Participants stated that the program was easy to use, helped them to learn about depression, taught them to self-monitor their mood, and the program was generally regarded as a source of support and as beneficial. However, participants did not use the online chat counseling.

### Study Protocols

The Rojas et al. (33) project aims to study the effectiveness of a technology-assisted training and supervision program to enhance depression management in primary care clinics in Santiago. In order to develop a comprehensive supervision program, trained administrative staff will contact patients from a call center to support treatment adherence, and psychiatrists will supervise the primary care team, using a web-based platform.

The Mascayano et al. (34) project aims to develop and evaluate an online intervention for preventing suicide and improving mental health among adolescents. The program uses a web-based platform and a mobile application to cultivate a virtual community. It includes both informational and interactive features, ranging from suicide prevention strategies (e.g., a chat with a psychologist), to components designed to increase interactions between participants and promote a sense of belonging and connection. During the intervention, two psychologists will monitor the platform, identifying behaviors associated with suicide risk and proceeding to an established emergency protocol. A group of adolescents played a role in the creation of the program and have access to the platform to facilitate discussions.

The Irarrázaval et al. (under review) project aims to implement a telepsychiatry service to enhance the provision of primary care mental health for children and adolescents living in substitute care facilities. Two primary care mental health teams, who provide mental health care to vulnerable children and adolescents, will receive bimonthly videoconference supervision from psychiatrists located at the Faculty of Medicine of the University of Chile over a 6-month period.

## Discussion

This mini-review shows that Chile has presented a slow but progressive development of e-mental health research. Nine studies that used Internet and digital technologies to address mental health are presented: three RCTs, three acceptability and feasibility studies and three on-going studies without published results. The interventions included are heterogeneous in terms of participants and contexts (e.g., adolescents, adults, patients, professionals, rural, urban), aims (e.g., collaborative care treatment, monitoring relapse prevention) and modalities (e.g., website, online games). This demonstrates the diversity of interventions that can be made through digital technologies to meet different needs. Despite this heterogeneity, the majority of the interventions reported in this study are meant to reduce the burden of depression, the most worrying mental health problem in Chile (16).

Studies have prioritized the use of digital technologies to assist vulnerable groups in low-resource settings, especially in primary care. This is especially relevant for low-resource health services located in rural hard-to-reach areas where there is no specialized mental health care nearby. Remote collaborative care and telepsychiatry interventions reviewed in this study show that it is feasible to use technologies for collaborative care in this particular context, with adequate acceptability and satisfaction levels among health care teams and patients.

The reviewed studies are addressing not only adult populations but also children and adolescents, who are in a critical period where many mental health disorders arise. Implementing Internet-based interventions to prevent or treat mental health problems in this age group can be a reasonable way of dealing with the severe scarcity of mental health care resources in developing countries, considering that this age group is familiar with digital technologies. The reviewed studies show that Internet-based interventions are feasible in children and adolescents, with adequate acceptability levels.

Despite the recent growth of this field, the number of conducted and on-going studies is still low when compared to developed countries. Most studies are mainly concentrated on the feasibility and acceptability of the interventions. The few effectiveness studies (RCTs) show that the interventions supported by digital technologies are feasible, but failed to demonstrate their effectiveness in reducing symptomatology. Demonstrating significant differences between interventions and control conditions is hindered by low sample sizes that lead to low statistical power. Therefore, there is a need to develop more effectiveness studies with larger sample sizes and substantial follow-up periods. Likewise, studies need to develop understanding on how interventions work, for whom, and why (mechanisms of change) they work. Given that one of the main arguments for introducing Internet-based interventions is their cost-saving potential, cost-effectiveness evaluations are also of crucial importance to the field (37).

Some of the reviewed studies reported particular concern for the social adaptation and cultural sensitivity of interventions, highlighting that the involvement of participants (patients and providers) in the design of interventions could be crucial for their success. For example, in the Mascayano et al. (34) study, the investigators included peer-adolescents in their research team as “experts by experience” to advise the creation of the intervention. Similarly, Carrasco (31) conducted focus groups with adolescents in order to get information for the design of their video game intervention. The Martínez et al. (under review) study also concluded that it is necessary to involve the target users in the design of the intervention in order to encourage participation.

The studies reviewed consistently show a gap between acceptability and usability/adherence of interventions. In this regard, future studies need to address challenges associated with high attrition in these programs. In the CTA program, in order to improve user retention, the authors suggested adding more personalized and interactive content, and more concrete tasks for users to perform during their interaction with it. Furthermore, in the evaluation of this program, Parada et al. (38) suggested using a persuasive systems design approach. Likewise, in the study of Espinosa et al. (32) participants who had completed the intervention suggested diversifying the monitoring assessments and having the possibility to interact with a therapist from the treatment center where they underwent face-to-face treatment. Both Martínez et al. (under review) and Espinosa et al. (32) suggested enabling peer communication. ASCENSO and CTA studies showed that email does not seem to be a good way to contact and monitor participants (27, 29). A possible way to improve the usability and effectiveness of e-mental health interventions is to facilitate access to the content of programs using mobile devices like smartphones (8, 9).

The case of ASCENSO and CTA reveals that it is probably relevant for participants to know that online personalized contact and support from a mental health professional is available if they require it. Similarly, a newer form of a blended care intervention that combines the strengths of Internet-based interventions and face-to-face therapy is increasingly being applied in mental health care (39). Even though blended care studies have not been conducted in Chile, this format is presented as a good alternative to address the growing need for access to psychological support and treatment for mental disorders in developing countries (13), without disproportionately increasing costs for health services.

A critical issue in mental health research is the gap between what is known about interventions and what is provided in daily care routines. To ensure the scaling-up of the interventions reviewed, it is necessary to produce methodological developments in effectiveness and cost-effectiveness studies, but also in implementation methods (40). Since a key strategy for increasing the use of digital technologies in mental health programs includes the development of translation-focused research, studies need to place greater emphasis on facilitators and (structural and cultural) barriers to the implementation of these technologies in the Chilean health system, especially in primary care services.

Most developing countries do not have sufficient mental health resources or highly trained professionals in mental health. The accelerated growth in mobile and Internet connectivity witnessed in the recent years have boosted the development of the e-mental health field in order to reduce this gap. The lessons learned in the Chilean context can provide local evidence for persuading policymakers and other stakeholders to support Internet-based interventions, which is critical to define them as a priority area for research and to ensure funding to widely disseminate and scale up these interventions. The Chilean case can also inspire initiatives in other developing countries.

## Conclusions

Internet-based interventions are at an early stage of development in Chile. There are few studies on effectiveness and no studies on cost-effectiveness, which makes it difficult to disseminate and scale up these interventions. However, the growing amount of knowledge accumulated in the Chilean context could guide practices in other developing countries for supporting the mental health of underserved populations.

## Author Contributions

GR, VM, PM, and ÁJ-M contributed conception and design of the study. GR, VM, PM, and ÁJ-M organized the database. ÁJ-M and PF wrote the first draft of the manuscript. All authors contributed to manuscript revision, read, and approved the submitted version.

### Conflict of Interest

The authors declare that the research was conducted in the absence of any commercial or financial relationships that could be construed as a potential conflict of interest.
