# Supplementary material for: Improving Mental Health Care in Developing Countries Through Digital Technologies: A Mini Narrative Review of the Chilean Case
Source: Front Public Health. 2019 Dec 20;7:391. doi: 10.3389/fpubh.2019.00391 (PMC6933524; doi:10.3389/fpubh.2019.00391)
Supplement: Supplementary file 1 [file Table_1.DOCX]

Supplementary Material

# Search strategies

**Pubmed**

Chile[Title/Abstract] AND (Mental Disorders[Mesh] OR Psychiatric Diagnosis[Title/Abstract] OR Behavior Disorder*[Title/Abstract] OR Mental Health[Mesh] OR Psychiatric Illness[Title/Abstract] OR Depression[Mesh] OR Drug Abuse[Title/Abstract] OR Drug Dependence[Title/Abstract] OR Depression[Title/Abstract] OR Anxiety[Title/Abstract] OR Bipolar[Title/Abstract] OR Schizophrenia[Title/Abstract] OR Alcohol[Title/Abstract] OR Drugs[Title/Abstract] OR "Substance Use"[Title/abstract] OR "Substance Abuse"[Title/Abstract]) AND (Acceptability[Title/Abstract] OR Effectiveness[Title/Abstract] OR Efficacy[Title/Abstract] OR Feasibility[Title/Abstract]) AND ("Information Science"[Mesh] OR Internet[Title/Abstract] OR Computer[Title/Abstract] OR Technolog*[Title/Abstract] OR Digital[Title/Abstract] OR Electronic[Title/Abstract])

**Embase**

Chile:ab,ti AND (‘mental disease’/exp OR ‘psychiatric diagnosis’/exp OR ‘mental health’/exp OR ‘psychiatric illness*’:ab,ti OR ‘depressive disorder*’:ab,ti OR ‘psychotic disorder*’:ab,ti OR ‘drug abuse’/exp OR ‘depression’:ab,ti OR ‘anxiety’:ab,ti OR ‘bipolar’:ab,ti OR ‘schizophrenia’:ab,ti OR ‘alcohol’:ab,ti OR ‘drugs’:ab,ti OR ‘substance use’:ab,ti OR ‘substance abuse’:ab,ti) AND (‘acceptability’:ab,ti OR ‘effectiveness’:ab,ti OR ‘efficacy’:ab,ti OR ‘feasibility’:ab,ti) AND (‘information science’/exp OR ‘internet’:ab,ti OR ‘computer’:ab,ti OR ‘technolog*’:ab,ti OR ‘digital’:ab,ti OR ‘electronic’:ab,ti)

**Scielo**

CHILE AND ((((((((((MENTAL HEALTH) OR (MENTAL DISORDERS)) OR PSYCHIATR$) OR PSYCHOLOG$) OR DEPRESSION) OR ANIXETY) OR BIPOLAR) OR SCHIZOPHRENIA) OR ALCOHOL) OR DRUG) AND ((((ACCEPTABILITY) OR EFFECTIVENESS) OR EFFICACY) OR FEASIBILITY) AND (((((INTERNET) OR COMPUTER) OR TECHNOLOG$) OR DIGITAL) OR ELECTRONIC)
